# Evaluation of *Staphylococcus aureus* Subtyping Module for Methicillin-Resistant *Staphylococcus aureus* Detection Based on Matrix-Assisted Laser Desorption Ionization Time-of-Flight Mass Spectrometry

**DOI:** 10.3389/fmicb.2019.02504

**Published:** 2019-10-31

**Authors:** Yanyan Hu, Yonglu Huang, Yangzi Lizou, Jiaping Li, Rong Zhang

**Affiliations:** ^1^Clinical Microbiology Laboratory, The Second Affiliated Hospital of Zhejiang University School of Medicine, Hangzhou, China; ^2^School of Laboratory Medicine and Life Science, Wenzhou Medical University, Wenzhou, China

**Keywords:** matrix-assisted laser desorption ionization time-of-flight mass spectrometry, methicillin-resistant *Staphylococcus aureus*, sensitivity, specificity, resistant

## Abstract

A recently developed *S. aureus* subtyping module for rapidly differentiate methicillin-resistant *Staphylococcus aureus* (MRSA) from methicillin-susceptible *S. aureus* (MSSA) had been introduced into China. The principle of this method was to identify the methicillin resistance through detection of a specific phenol soluble modulin-mec peak (PSM-mec) by matrix-assisted laser desorption/ionization time of flight mass spectrometry (MALDI-TOF MS). A total of 347 non-duplicated *S. aureus* strains were collected from the Second Affiliated Hospital of Zhejiang University School of Medicine during January 2014 to February 2019. The sensitivity, specificity, positive predictive value (PPV), and negative predictive value (NPV) of the automated subtyping module in identifying MRSA were evaluated. The specificity and PPV of this method were both 100%, and the sensitivity was 60.2%. PSM-bearing MRSA was reported with different prevalence from different parts of the world, our sample collection has the highest percentage so far. The repeatability showed that 1.7% (6/347) and 18.4% (64/347) were reported differently in the intra- and inter-batch analysis, respectively, which demonstrated that the threshold of this method could be further optimized to increase the sensitivity of MRSA detection. Overall, Bruker^™^ MALDI Biotyper can detect *S. aureus* isolates with a quite high specificity and expedite the identification of MRSA isolates without using extra reagent, labor, or time. The reduced turnaround time of MRSA identification is essential for appropriate therapeutic management and timely intervention for infection control.

## Introduction

*Staphylococcus aureus*, one of the most important and prevalent nosocomial pathogens, often causes a wide range of infections involving skin, blood, endocarditis, or pneumonia (Tong et al., 2015). Of particular concern is the emergence of methicillin-resistant *S. aureus* (MRSA) strains. Infections caused by MRSA had a higher morbidity and mortality compared with those of methicillin-susceptible *S. aureus* (MSSA) (Cosgrove et al., 2003). For MRSA, all the currently available beta-lactam antimicrobial agents with the exception of the new generation cephalosporins are limited for therapy, vancomycin, or the other antibiotics should be chose for treatment (Liu et al., 2011). Given the substantial morbidity and mortality associated with MRSA and the limitation scheme of treatments, it is necessary to develop new drugs as well as shorten the time for MRSA identification.

At present, there are mainly four methods for MRSA identification including traditional culture and susceptibility testing, chromogenic media, real-time polymerase chain reaction, and analysis of the mass spectra based on the ClinPro Tools software. Each has its own advantages and disadvantages (Palavecino, 2014; Wang et al., 2018). Rhoads et al. had developed another rapid detection method of MRSA based on the specific phenol soluble modulin-mec peak (PSM-mec) detection by matrix-assisted laser desorption ionization time-of-flight mass spectrometry (MALDI-TOF MS) (Rhoads et al., 2016). The PSM-mec peptide is encoded by staphylococcal cassette chromosome mec (SCCmec) types II, III, and VIII in the vicinity of *mecA* (Chatterjee et al., 2011). Whereas Timke et al. (2017) had developed a *S. aureus* subtyping module for MRSA identification based on the PSM-mec peak as an enhanced function to the Bruker MALDI Biotyper system without any extra manual operation, time, or reagent. Till now, only Germany, Canada, and the United States had reported the specificity and sensitivity data of this method in the identification of MRSA (Budvytiene et al., 2016; Rhoads et al., 2016; Lagacé-Wiens et al., 2017; Timke et al., 2017), and the detection rate of such *S. aureus* varies in different regions (approximately 10–30%). PSM data of such PSM-mec peak detection in China and in various specimens were limited. Thus, in the current study, we intended to evaluate the sensitivity, specificity, and repeatability of the automatic *S. aureus* subtyping module for the first time and to observe the proportion of such *S. aureus* in China.

## Materials and Methods

### Bacterial Identification and Antimicrobial Susceptibility

About 347 non-duplicated *S. aureus* were collected from the Second Affiliated Hospital of Zhejiang University, School of Medicine during January 2014 to February 2019 including feces (*n* = 163), sputum (*n* = 94), blood (*n* = 15), and sterile body fluid (*n* = 75). Species identification and antimicrobial susceptibility were first performed using the VITEK-2 compact system (bioMérieux, Marcy-l’Étoile, France) and interpreted in accordance with the Clinical and Laboratory Standards Institute Guideline document M100. Then we identified the species by MALDI-Biotyper (Bruker Daltonics, Billerica, MA, USA) and tested antimicrobial susceptibility by disk diffusion method of cefoxitin (30ug). Cefoxitin is tested as a surrogate for oxacillin in disk diffusion method. Isolates that test resistance by cefoxitin disk should be reported as oxacillin resistant. *S. aureus* ATCC 25923 and *S. aureus* ATCC43300 (MRSA) were used as quality control. The results were interpreted according to the criteria of CLSI M100-S28 (CLSI, 2018).

### Methicillin-Resistant *Staphylococcus aureus* Detection by Matrix-Assisted Laser Desorption Ionization Time-of-Flight Mass Spectrometry

All the isolates were recovered from the frozen storage onto the blood agar plates for 24 h at 35°C. Then the following steps were achieved the same as routine clinical operation. First, a fresh single bacterial colony was picked up and directly smeared onto a MALDI steel 96-spot target plate. Second, 1 μl of 70% formic acid was added to each target spot and dried at room temperature. Third, 1 μl of a-cyano-4-hydroxycinnamic acid (CHCA) matrix solution was introduced on the target spot again and air dried before analyzing on the MALDI-TOF MS. Mass spectrum was acquired and analyzed using a MALDI Biotyper standard system equipped with MBT Compass software (Bruker Daltonics GmbH, Bremen, Germany). For each sample, 240 laser shots were collected using the automatic mode, and *Escherichia coli* ATCC8739 was used as control in calibration of the instrument. Mass spectrum of each isolate was compared with those in the database and assigned scores. *S. aureus* strains with log (scores) ≥2.0 were further automatically identified by the subtyping module. When the peak of 2413 ± 2 m/z exists, the software alerts the *S. aureus* strain as “presumptive PSM positive MRSA”, otherwise, only *S. aureus* was reported (Supplementary Figure S1). For the repeatability analysis, each strain was identified thrice on each individual clone to evaluate the inter-batch repeatability of this method. Simultaneously, mass spectra of the first batch samples were acquired twice on the same spot to evaluate the intra-batch repeatability.

### Data Analysis

The Flex Analysis 3.3 program (Bruker Daltonics GmbH, Bremen, Germany) was used for the spectrum analysis. All the spectra were smoothed and baseline subtracted before comparing of the specific PSM-mec peak (2,413 ± 2 m/z). ClinProTools software (version 3.0, Bruker Daltonik GmbH, Bremen, Germany) was used to analyze the discrepancy between presumptive PSM positive MRSA (PSM-MRSA) and MSSA isolates, PSM-MRSA and MRSA isolates without the specific PSM-mec peak [MRSA(−)], and those strains with inconsistent results during the experiment. MBT Compass Explorer software (Bruker Daltonik GmbH, Bremen, Germany) was performed to show normalized spectra in gel view.

### Statistics Analysis

To evaluate the performance of MRSA detection using MALDI-TOF MS *S. aureus* subtyping module, we use sensitivity, specificity, positive predictive value (PPV), and negative predictive value (NPV) as metrics. Pearson chi-square (*χ*^2^) test or Fisher’s exact test was used to compare the sensitivity discrepancy of various specimen types and different batches. *p* < 0.05 was considered to be significantly different. Statistic analysis was performed using the Statistic Package for Social Sciences Version 23.0 (SPSS Inc., Chicago, IL, USA).

## Results

Bacterial identification and cefoxitin antimicrobial susceptibility confirmed that 241 strains were MRSA and 106 were MSSA (Table 1). All the 347 isolates were identified as *S. aureus* with log (scores) ≥2.0. Among which, #121, #126, and #120 isolates were identified as “presumptive PSM positive MRSA” for batch #1, #2, and #3, respectively (Table 1). The sensitivity of each batch was 50.2, 52.3, and 45.6%, respectively. After combining the results of the three batches, the sensitivity of this method reached to 60.2%. Sensitivities of various specimen types were also different. However, the specificity was 100% despite of the batch and specimen type (Table 1). Meanwhile, there was no difference in sensitivity among the specimen type groups (*χ*^2^ = 2.224, *p* = 0.329) or the batch groups (*χ*^2^ = 3.778, *p* = 0.286).

**Table 1:** Statistics analysis of different groups of *S. aureus* strains.

	*S. aureus* (No.)	MRSA (No.)	MSSA (No.)	PSM-MRSA (No.)	PPV^*^ (%)	NPV^#^ (%)	Sensitivity (%)	Specificity (%)
Batch 1	347	241	106	121	100.0	46.9	50.2	100.0
Batch 2	347	241	106	126	100.0	48.0	52.3	100.0
Batch 3	347	241	106	110	100.0	44.7	45.6	100.0
Total^a^	347	241	106	145	100.0	52.5	60.2	100.0
Feces	163	153	10	88	100.0	13.3	57.5	100.0
Sputum	94	46	48	27	100.0	71.6	58.7	100.0
Sterile body fluid	75	34	41	23	100.0	78.9	67.7	100.0
Blood	15	8	7	7	100.0	87.5	87.5	100.0

* PPV, positive predictive value;

# NPV, negative predictive value;

a *The data listed in the row “total,” “feces,” “sputum,” “sterile body fluid,” and “blood” combined the three batches of “PSM-MRSA” isolates. The isolate were judged as “PSM-MRSA” as long as one of the batches was identified as “PSM-MRSA” by MALDI Biotyper*.

**Figure 1:**
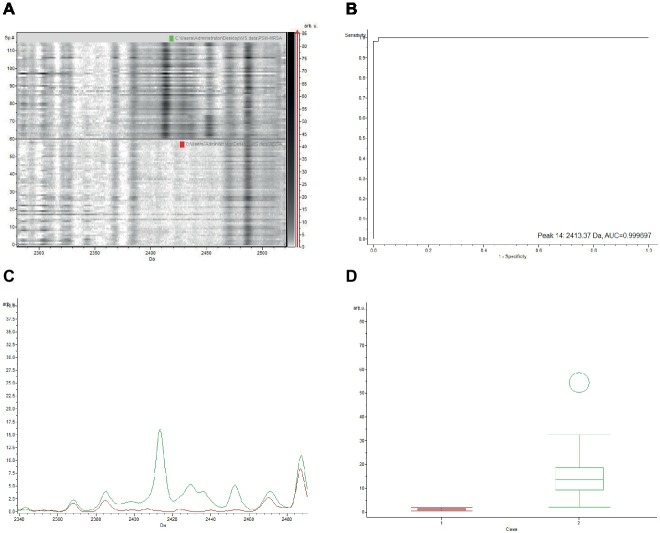
Discrepancy of the characteristic 2,413 m/z peak between PSM-MRSA and MSSA classification models. **(A)** The gel view of the respective section. The red arrow indicates the characteristic peak of 2,413 m/z. Intensities of peaks were expressed in arbitrary units (arb.u.). **(B)** The AUC of the 2,413 m/z peak. **(C)** Average spectra of 2,413 m/z peak. Green line and red line represent for PSM-MRSA and MSSA group, respectively. **(D)** Display of single peak variance of 2,413 m/z.

**Figure 2:**
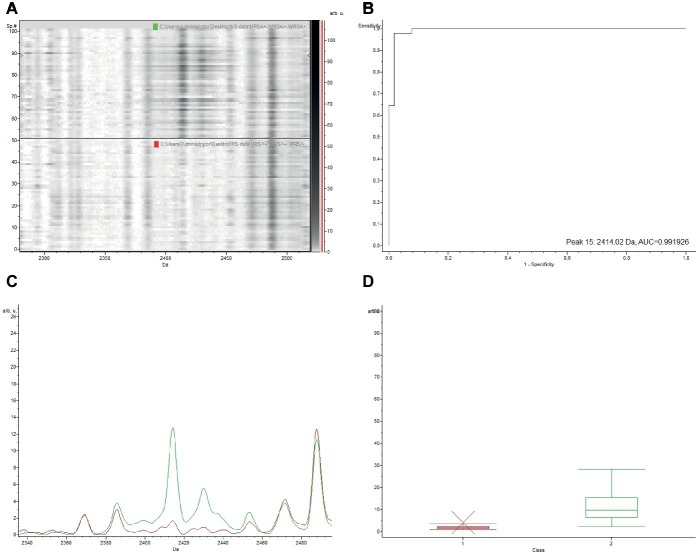
Discrepancy of the characteristic 2,413 m/z peak between PSM-MRSA and MRSA(−) classification models. MRSA(−) represents for MRSA isolates without the specific PSM-mec peak. **(A)** The gel view of the respective section. The red arrow indicates the characteristic peak of 2,413 m/z. Intensities of peaks were expressed in arbitrary units (arb.u.). **(B)** The AUC of the 2,413 m/z peak. **(C)** Average spectra of 2,413 m/z peak. Green line and red line represent for PSM-MRSA and MRSA(−) group, respectively. **(D)** Display of single peak variance of 2,413 m/z.

The gel view representation of PSM-MRSA and MSSA classification models revealed a significant discrepancy at the peak of m/z 2,413 (Figure 1A). The area under curve (AUC) of the m/z 2,413 peak was 0.9997 (Figure 1B). The average intensity and single peak variance of the m/z 2,413 peak of the two classification models are further illustrated in Figures 1C,D. Similarly, if we compare the two classification models of PSM-MRSA and MRSA(−), the 2,413 m/z peak also demonstrated significant discrepancy between the two classes (Figure 2). The AUC of the specific peak was 0.992 (Figure 2B). These results demonstrated the specific characteristics and patterns of m/z 2,413 peak in the different classifications. Specifically, the classification of the PSM-MRSA and MSSA showed a better satisfactory separation at peak 2,413 than the classification of the PSM-MRSA and MRSA(−) isolates.

**Figure 3:**
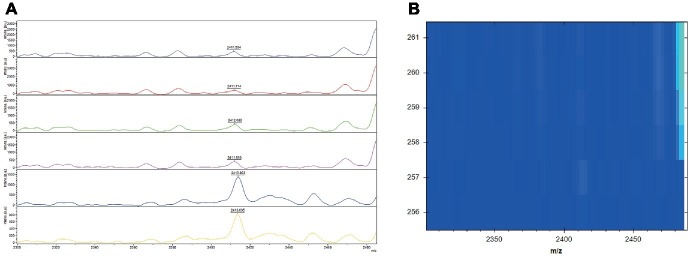
Mass spectra of the characteristic 2,413 m/z peak in the intra batch repeatability and their corresponding normalized spectra in gel view. **(A)** Mass spectra of the characteristic 2,413 m/z peak analyzed by flexanalysis. The red rectangle displayed peaks at 2,413 2 m/z of the three isolates. (+) and (−) represents for PSM-MRSA and MRSA(−), respectively. x axis, mass per charge in daltons (m/z, Da); y axis, absolute intensity of signal. **(B)** Normalized spectra in gel view. The red rectangle displayed the corresponding peaks at 2,413 2 m/z of the three isolates.

For the intra-batch repeatability, 1.7% (6/347) was identified differently. Three of the strains were chosen randomly for normalized spectra analysis. We can obviously found that all the strains had a peak at 2413 m/z (Figure 3A); however, when the peaks were normalized, only those with relatively high intensities were identified as PSM-MRSA (Figure 3B). Likewise, the normalized spectra analysis of the 18.4% (64/347) isolates with inconsistent results during the inter-batch repeatability analysis were in accordance with the above strains (data not shown).

## Discussion

MALDI-TOF MS had been mainly used in many clinical microbiology laboratories for effective and rapid identification of bacterial species (Ge et al., 2017). It also provides an alternative solution for molecular typing (Spinali et al., 2015) and antibiotic resistant study (Oviano and Bou, 2019). It was first applied to differentiate MRSA from MSSA by intact cell mass spectrometry in the year of 2000 (Edwards-Jones et al., 2000). In the current study, a recently developed *S. aureus* subtyping module was evaluated for MRSA detection based on MALDI-TOF MS. Unlike the previously reported parallel method (Rhoads et al., 2016), this subtyping module do not need any extra operations or a third party software assistance. It could identify MRSA automatically through the bacteria identification process.

Our results showed a quite high specificity of this method, which is accordant with researches in Germany, Canada, and the United States, and no false positive strains were detected among the *S. aureus* subtyping module evaluations (Budvytiene et al., 2016; Rhoads et al., 2016; Lagacé-Wiens et al., 2017; Timke et al., 2017). The sensitivity (60.2%) of this study was much higher than previously reported (Budvytiene et al., 2016; Rhoads et al., 2016; Lagacé-Wiens et al., 2017; Timke et al., 2017) which might indicate the prevalence of the SCCmec types II, III, and VIII strains in China is higher than Europe and America. However, we have to point out that the SCCmec types of these clinical MRSA isolates were not evaluated in our study.

Clinpro Tools analysis demonstrated that the 2,413 m/z peak could commendably distinguish MRSA from MSSA, as well as PSM-MRSA from MRSA(−). However, for the 1.7 and 18.4% inconsistent results in the intra- and inter-batch repeatability analysis, we speculate that some strains of the MRSA(−) group might possess the 2,413 m/z peak with relatively low intensity in view of the slight difference of the above two classification groups in AUC (0.9997 vs. 0.9992) and the very weak peak of 2,413 m/z in the MRSA(−) group rather than the MSSA group. As expected, the results of Figure 3 proved this. Only the relatively high intensity of the normalized spectra was reported as PSM-MRSA, nevertheless, those possess the 2,413 m/z peak but with relatively low intensity strains were not reported. The inconsistent results demonstrated that the threshold of this method might be a little high and could be further optimized to increase the sensitivity of MRSA detection. Meanwhile, in order to achieve optimization, the specificity should be monitored synchronously when adjusting parameters. However, it might be better to have 100% specificity than dealing with false positives.

To our knowledge, this present study is the first using a MALDI-TOF MS-based *S. aureus* subtyping module to differentiate MRSA from MSSA in China. In addition, previous studies did not distinguish between specimen types. Our study evaluated the sensitivity discrepancy of various specimen types using the *S. aureus* subtyping module for the first time. The result revealed that this subtyping module might be suitable for *S. aureus* strains collected from all kinds of specimens and the prevalence of the SCCmec types II, III, and VIII strains of different specimens might have no statistical difference in China. Meanwhile, this research also seems to be the first study to evaluate the repeatability of this method, which explains why we found that this method could be further optimized.

In conclusion, Bruker^™^ MALDI Biotyper can detect the *S. aureus* isolates with a high specificity and expedite the identification of MRSA isolates without adding any reagent, labor or time which is essential for appropriate therapeutic management and timely intervention for infection control.

## Data Availability Statement

All datasets generated for this study are included in the article/Supplementary Material.

## Ethics Statement

The study was approved by the Ethics Committee of Second Affiliated Hospital of Zhejiang University, School of Medicine (2017-099). All subjects gave written informed consent in accordance with the Declaration of Helsinki.

## Biosafety Statement

All concerns related to the safe and appropriate use of human-derived materials, infectious agents, or genetically modified organisms were approved by the Institutional Biosafety Committee of Second Affiliated Hospital of Zhejiang University, School of Medicine. All experiments were conducted under the guidelines from the Biological Agent Reference Sheet.

## Author Contributions

RZ and YaH designed the study. YL and YoH did the experiment. YaH and JL analyzed and interpreted the data. YaH and RZ wrote the manuscript. All authors read and approved the final manuscript.

### Conflict of Interest

The authors declare that the research was conducted in the absence of any commercial or financial relationships that could be construed as a potential conflict of interest.

## Supplementary Material

The Supplementary Material for this articles can be found online at: https://www.frontiersin.org/articles/10.3389/fmicb.2019.02504/full#supplementary-material
