# Supplementary material for: Evaluation of Staphylococcus aureus Subtyping Module for Methicillin-Resistant Staphylococcus aureus Detection Based on Matrix-Assisted Laser Desorption Ionization Time-of-Flight Mass Spectrometry
Source: Front Microbiol. 2019 Oct 31;10:2504. doi: 10.3389/fmicb.2019.02504 (PMC6834645; doi:10.3389/fmicb.2019.02504)
Supplement: Supplementary file 1 [file Data_Sheet_1.PDF]

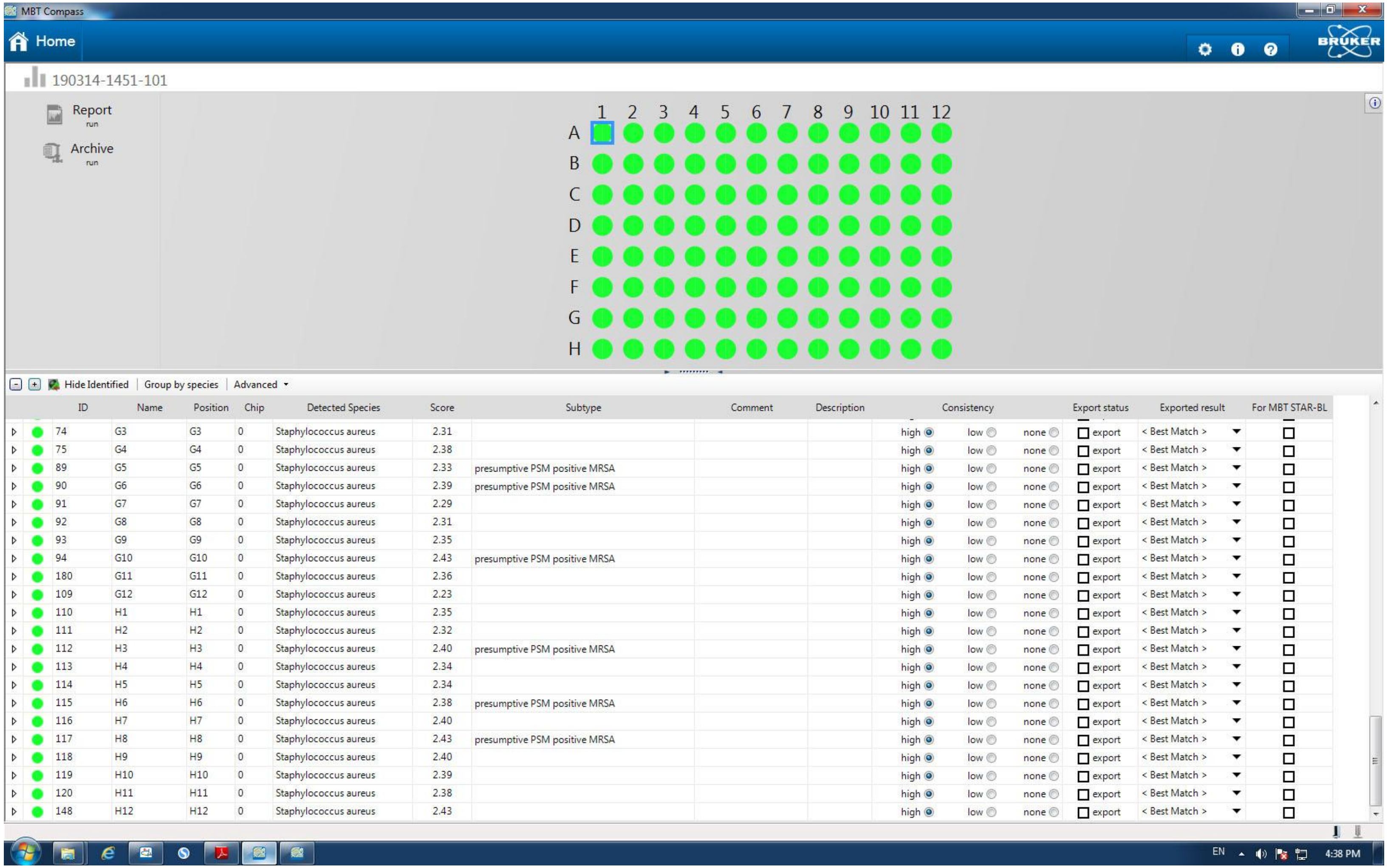


**Fig. S1.** MALDI Biotyper software screenshot with PSM negative and PSM positive *S.aureus* identification results.
